# Engaging Expectant and Parenting Adolescents: Lessons from the Massachusetts Pregnant and Parenting Teen Initiative

**DOI:** 10.1007/s10995-020-02880-z

**Published:** 2020-01-24

**Authors:** Justine Egan, Nazmim Bhuiya, Lissette Gil-Sanchez, Stephanie Campbell, Jill Clark

**Affiliations:** 1 Office of Sexual Health and Youth Development, Massachusetts Department of Public Health, 250 Washington St, Boston, MA 02108 USA; 2 Harvard T.H. Chan School of Public Health, 677 Huntington Ave, Boston, MA 02115 USA; 3 Massachusetts Department of Public Health, 250 Washington St, Boston, MA 02108 USA; 4 Office of Sexual Health and Youth Development, Massachusetts Department of Public Health, 250 Washington St, Boston, MA 02108 USA; 5 Division of Child/Adolescent Health and Reproductive Health, Massachusetts Department of Public Health, 250 Washington St, Boston, MA 02108 USA

**Keywords:** Adolescent parents, Youth engagement, Young families

## Abstract

**Introduction:**

Programs supporting adolescent parents have been shown to increase socio-economic opportunities and promote healthy child development for young families, but retaining young parents is challenging. The Massachusetts Pregnant and Parenting Teen Initiative (MPPTI) offers case management and linkages to community and clinical services to young families. We examine engagement strategies identified by MPPTI participants and staff members in relation to participant retention by program site to identify potential strategies for increasing program engagement.

**Methods:**

We employed a mixed-methods approach incorporating quantitative data on program participant characteristics and program retention by site with qualitative data from staff and participant interviews and focus groups.

**Results:**

Key program engagement strategies identified by both MPPTI staff and youth participants were social-emotional supports, staffing model, and concrete supports. We found significant differences in program retention by site; the two sites with the highest levels of program retention offered all engagement strategies identified.

**Discussion:**

Quantitative data on program retention coupled with qualitative data from staff and youth interviews suggests that in our program, there may be an association between the engagement strategies identified and levels of program retention.

## Significance

The majority of research on youth program engagement focuses on youth as a broad group and not specifically on adolescent parents. This paper builds upon previous research by identifying specific strategies for engaging high-risk young families in case management programming. The effect of these strategies on program retention and subsequent program outcomes should be further explored.

## Introduction

Unplanned adolescent births are associated with decreased socioeconomic opportunities and poor health outcomes for both the adolescent parents and their children. Adolescent parents more frequently report postpartum depression (Lanzi et al. 2009; Reid and Meadows-Oliver 2007), have low levels of social and community support (Angley et al. 2015; Crawford et al. 2011; Ellis-Sloan and Tamplin 2019; Logsdon et al. 2005), have higher incidences of poor birth outcomes (Malabarey et al. 2012), and are more likely to have lower educational attainment and employment levels (Assini-Meytin and Greene 2015) compared to older parents. Children of teen parents are more likely to have lower educational achievement and income levels later in life (Lipman et al. 2011).

The Massachusetts Pregnant and Parenting Teen Initiative (MPPTI) offers case management and linkages to community and clinical services to expectant and parenting adolescents aged 14–24 years. The goal of MPPTI is to provide a comprehensive support model for expectant and parenting adolescents so that they (1) increase academic achievement and employment, (2) improve reproductive health outcomes, and (3) enhance family stability. MPPTI uses a two-generation model consisting of the following core services: helping participants set and work toward achieving education/employment goals; assessing reproductive, primary, and pediatric care needs and providing supported referrals; providing behavioral health supports as necessary either directly or via referral; assisting with securing housing; conducting screenings for child development and providing referrals to early intervention services; offering concrete supports such as child care and transportation; and providing health education. MPPTI targets adolescents who are disengaged from or ineligible for other young parent programming. Many programs serving young families have eligibility requirements such as serving only first-time parents or employing age cutoffs (i.e. serving only adolescents under age 21). Some programs may also restrict eligibility to participants who can commit to proscribed attendance requirements up front. To achieve program outcomes, MPPTI aims to engage participants in the program for a minimum of six months and ensure that participants have at least monthly check-ins with program staff.

During 2014–2017, services were provided by community-based agencies in five Massachusetts communities with high teen birth rates. The funded communities—Chelsea, Holyoke, Lawrence, New Bedford, and Springfield—have teen birth rates that are 3 to 4 times the state rate and up to 1.7 times the national rate (Massachusetts Department of Public Health 2018; Martin et al. 2018). Although core services were the same across all five agencies, the service delivery approach differed. Four of the five agencies used a “centralized” staffing model where services were offered by the agency and referrals were made for services not directly provided. Under this model, participants were assigned to a youth worker who acted as the participant’s point person. One agency used a “network” model where one organization served as the backbone and subcontracted with multiple organizations to provide direct services. Participants typically did not have one point person and interacted with multiple staff at different agencies.

Programs supporting adolescent parents have been shown to increase socioeconomic opportunities and promote healthy child development (Jacobs et al. 2016; Ickovics et al. 2016), but previous studies have identified barriers to program engagement such as transportation and child care (Asheer et al. 2014). Negative experiences with health care providers and/or fear of being judged or stereotyped by providers may also impact youth seeking or remaining engaged in services (Harrison et al. 2017; Recto and Champion 2018). Due to these barriers, retaining young parents most in need is challenging (Asheer et al. 2014).

Strategies that have been associated with higher levels of youth engagement include meaningful staff-participant relationships and quality program content (Greene et al. 2013), whereas the impact of financial incentives on increasing youth engagement is mixed (Greene et al. 2013; Gneezy et al. 2011). The majority of research on youth program engagement focuses on youth as a broad group and not specifically on adolescent parents. Many adolescent parents experience postpartum depression and/or social isolation (Angley et al. 2015; Crawford et al. 2011; Ellis-Sloan and Tamplin 2019; Logsdon et al. 2005) and therefore may have unique needs for social support compared to other youth populations. The role that social-emotional supports may play in program engagement for adolescent parents has been largely unexplored in the literature. The aim of this study is to build upon existing research identifying strategies for increasing participant engagement that may be useful to other programs serving young families. We examined participant retention by program site in relation to engagement strategies identified by program participants and staff members to identify potential strategies for increasing program engagement among young families.

## Methods

To understand the relationship between engagement strategies employed by program staff, youth perception of the program, and program retention, we employed a mixed-methods approach. We used quantitative methods to describe participant characteristics and examine differences in program retention by agency. Qualitative data were used to understand what was helpful from youth and staff’s point of view for increasing program engagement.

We measure program retention by the number of months participants were actively engaged in the program. Participants are considered “active” if they have, at a minimum, monthly check-ins with program staff to receive case management services. Many participants receive case management services more than once per month and participate in other programmatic activities such as attending support groups, attending parenting classes, and accessing health services. A preliminary analysis of program outcomes found that participants who were retained in the program for six months or longer were significantly more likely to achieve outcomes such as securing full or part-time employment and switching to a long-acting reversible contraceptive method. Participants that were retained for nine months or longer were more likely to secure stable housing, but the increase was not significant. Although length of time in the program does not provide information on the breadth of services received or program satisfaction, we use retention as a proxy for engagement.

Themes that emerged from youth focus groups and staff interviews were used to identify key strategies for program engagement. During staff interviews, we also identified services offered, such as social activities or wellness groups that differed from or went beyond core services. We examined levels of program retention by site in relation to the presence of the engagement strategies identified to draw preliminary conclusions about potentially effective strategies.

### Quantitative Data

Quantitative data on MPPTI participants served during 2014–2017 were collected by funded community agencies and updated monthly in a Microsoft Access database. Data collected included participant demographics, length of time in the program, and services received. Data were exported from Microsoft Access to SAS 9.3. SAS was used to generate descriptive statistics on participant characteristics and the number of participants recruited and retained in the program by site. A one-way analysis of variance (ANOVA) test was performed to determine if the mean number of months of program participation differed significantly by agency. Post hoc analyses comparing pairwise differences in agencies’ mean number of months of engagement were made using Scheffe’s test. Spearman correlation was used to test if there was any correlation between: (1) program engagement and participant characteristics, including demographics, being homeless, and education/employment status at the time of program entry, and (2) program engagement and number of engagement strategies employed by agency. Engagement strategies were coded as yes/no for the presence of the following strategies identified during qualitative interviews: support groups, social activities, and concrete supports.

### Qualitative Data

#### Program Staff Interviews

Key informant interviews were conducted with staff at each of the five community agencies implementing MPPTI. Five semi-structured group interviews lasting approximately one hour each were conducted with 16 staff members. Staff members were recruited for participation based on their roles in the program: at least one youth worker and one program coordinator or supervisor were invited to participate via email. Staff members were interviewed on the following topics using a 10-question guide: services provided, barriers to service delivery, approaches for recruiting and retaining participants, partnerships, resources/funding, successes, and lessons learned. We report only on services provided and participant recruitment and retention approaches. Staff interview results are reported by agency and not by individual staff member.

#### Focus Groups with Adolescent Parents

Six youth focus groups were conducted in English and Spanish with a convenience sample of 19 MPPTI participants across the five program sites. Current participants ages 18 and older were recruited by youth workers at their respective program sites to participate in the focus groups and were offered a meal and child care as incentives. Youth self-selected to participate after being provided with the information by youth workers. One focus group was conducted per community in four of the program sites; two focus groups were conducted in the community of Springfield. All focus group participants were between 18 and 24 years old. Sixteen of the participants identified as female and three identified as male. While the gender make-up of focus group participants was reflective of all program participants, youth under the age of 18 were not represented and information on participants’ race/ethnicity and specific needs, such as housing and academic supports, was not examined. Focus group length ranged from 30 to 60 minutes. A 12-question focus group guide was used; these questions covered motivation for participating in the program, services received, interaction with program staff, program satisfaction, and suggestions for improvement.

Focus groups and key informant interviews were conducted from July to August 2016. All participants consented to being interviewed and were able to opt out of answering questions they did not feel comfortable with. The Massachusetts Department of Public Health (MDPH) and Harvard University institutional review boards approved the project in July 2016. A Harvard University doctoral student with a master’s degree in public health and more than five years’ experience in qualitative data collection and analysis developed the question guides for the staff interviews and youth focus groups, conducted the interviews and focus groups, coded data, and led the qualitative data analysis. The question guides were not piloted before use. An MDPH staff member sat in on the interviews and focus groups, which were audio recorded and transcribed. Interviews and focus groups were held at the program sites. An inductive analysis approach (Elo and Kyngas, 2008) was used to identify and code key themes that emerged from focus groups and staff interviews. Analysis was conducted and a codebook was created using ATLAS.ti software. This codebook was used to group and quantify key themes. The COREQ checklist was used to guide qualitative data collection and reporting (Tong et al. 2007).

## Results

### Quantitative Results

During the period of 2014–2017, MPPTI served 588 participants; the number of participants served by each agency ranged from 86 to 150 (Table 1). There were some differences in youth served by agency: the Holyoke agency had higher percentages of participants reporting being homeless and/or not in school and not employed at program entry and being aged 18 years or older, whereas the Chelsea agency had a higher percentage of participants without a high school diploma, requiring an interpreter, and who were postpartum at program entry. Participant race/ethnicity also varied by agency (Table 1).

**Table 1:** MPPTI participant characteristics at program entry by site, 2014–2017

	Chelsea	Holyoke	Lawrence	New Bedford	Springfield	All agencies	Chi-Sq measures of independence
Female (%)	100.0	95.4	92.0	84.9	87.5	92.1	X2 (4) = 20.5, p = .0004, n = 579
Male (%)	0.0	4.6	8.0	15.1	12.5	7.9	
14–17 years (%)	16.7	2.8	20.0	32.9	17.1	17.5	X2 (8) = 32.9, p = < .0001, n = 572
18–19 years (%)	24.5	29.3	20.0	22.4	27.9	24.6	
20–24 years (%)	58.8	67.9	60.0	44.7	55.0	57.9	
Black NH (%)	5.6	12.0	2.7	12.8	12.9	8.7	X2 (12) = 47.2, p = < .0001, n = 565
Hispanic (%)	84.1	75.9	86.5	58.1	72.4	76.8	
White NH (%)	8.4	11.1	6.7	24.4	6.9	10.6	
Other NH (%)	1.9	1.0	4.1	4.7	7.8	3.9	
Pregnant (%)	7.5	75.9	69.1	40.7	75.8	26.6	X2 (4) = 31.8, p = < .0001, n = 578
Postpartum (%)	92.5	24.1	30.9	59.3	24.2	73.4	
Homeless (%)	13.2	35.6	3.5	5.8	20.7	19.6	X2 (4) = 58.4, p = < .0001, n = 552
Requires interpreter (%)	50.5	6.6	18.3	8.1	11.0	19.1	X2 (4) = 90.5, p = < .0001, n = 554
Not in school and not employed (%)	49.0	62.6	33.0	29.1	44.3	43.5	X2 (4) = 40.1, p = < .0001, n = 543
Did not graduate HS (%)	88.6	56.7	36.8	67.4	74.6	63.8	X2 (4) = 78.7, p = < .0001, n = 552

*Notes.* The number of participants varies for each characteristic due to missing data for some participants for some characteristics. The number of participants for each agency ranges from 86 to 150. *MPPTI* Massachusetts Pregnant and Parenting Teen Initiative; *NH* non-Hispanic; *HS* high school

Participant engagement and key engagement strategies varied across agencies (Table 2). All agencies offered support groups and concrete supports; three agencies offered social activities. The mean number of months that participants remained in the program differed by site as determined by one-way ANOVA (F [4, 565] = 13.12, *p* < 0.0001) and ranged from 9.6 to 18.8 months (Table 2). Post-hoc comparisons using Scheffe’s test demonstrated significantly different mean months of engagement only between certain agencies (Appendix).

**Table 2:** Program engagement strategies and program retention by MPPTI site, 2014–2017

Site	Support groups	Social activities	Concrete supports	Staffing model	Mean number of months of program participation
Chelsea	X	X	X	Centralized	18.8
Holyoke	X		X	Centralized	12.2
Lawrence	X	X	X	Centralized	15.1
New Bedford	X	X	X	Centralized	12.7
Springfield	X		X	Network	9.6

*Note. MPPTI* Massachusetts Pregnant and Parenting Teen Initiative

Spearman correlation indicated a significant positive association between number of months of program engagement and the following: number of engagement strategies (rs = 0.29, p < 0.0001), being Hispanic (rs = 0.16, p = 0.0003), requiring an interpreter (rs = 0.15, p = 0.001), and being female (rs = 0.12, p = 0.009). There were significant negative associations between number of months of program engagement and being homeless at the time of program entry (rs =  − 0.19, p < . 0001) and not being in school and not being employed at the time of program entry (rs =  − 0.13, p = 0.004). There were no significant associations between number of months of program engagement and age, having graduated high school, or pregnancy status.

### Qualitative Results

All staff from agencies interviewed stated they received a high number of referrals and had significant need for services for adolescent parents in their communities. Staff from all five agencies also reported that getting participants in the door and keeping them engaged were significant challenges. A staff member at one agency said,

Working with young people…can be difficult. They move a lot, their phones aren’t working anymore, they’re homeless, they’ve got stuff going on and they can’t be in one place long enough with you emotionally to connect with something. A lot of our folks have experienced multiple traumas in life and find trust very difficult. And the work is challenging.

Program components identified as being helpful by both the key informant interviews and the youth focus groups were (1) social-emotional supports, (2) staffing model, and (3) concrete supports. A summary of these key themes is presented in Table 3.

**Table 3:** Themes and findings from key informant interviews and youth focus groups

Theme	Key informant agencies (n = 5)	Youth participants (n = 19)
Social-emotional supports	Transformational, strengths-based, and trusting relationships with staff are key to program engagement. (n = 5) Offer a variety of activities, such as parenting groups, art therapy, and wellness groups to engage participants. (n = 2)	Good relationships with staff are the reasons participants continue to show up for programming. (n = 7) Social activities (e.g., field trips) are engaging aspects of the program. (n = 6) Support groups and opportunities to interact with other participants are key to the program. (n = 5) More social activities would improve program engagement. (n = 2)
Staffing model	Prioritizing staff team meetings to coordinate care allows for a better participant experience. (n = 2) Without consistent staff communication regarding service coordination, it becomes confusing about who is doing what. (n = 1)	Staff turnover is confusing in terms of coordinating referrals and whom to contact/who is contacting participants. (n = 2)
Concrete supports	Offer supports, such as child care, transportation, gift cards, bus passes, transportation, food, and diapers. (n = 5)	Concrete supports are a helpful part of the program. (n = 5)

### Social-Emotional Supports

Social-emotional supports such as supportive relationships with staff members, social activities (family activities or “field trips”), and support groups (wellness or parenting groups) were most frequently mentioned by youth as their reasons for remaining in the program. Program participants varied in their preferences for which types of social-emotional supports were most helpful. Some participants emphasized close relationships with program staff. Several participants preferred field trips or gatherings where the purpose was to have fun rather than formal support groups. Although many participants reported support groups to be engaging, some participants said they did not like groups.

*Supportive Staff Relationships.* The most common theme across both the key informant interviews and youth focus groups was creating trusting relationships between staff members and program participants. Program staff emphasized that building relationships with participants was key to engaging them in the program and getting them enrolled in direct services such as educational programs and reproductive health care. A program director stated, “I think the backbone of the model is to establish a transformational relationship between the youth worker and the mom.”

Youth spoke positively of program staff that were nonjudgmental and relatable because they had similar lived circumstances or prior experience working with youth: “With [staff X], I’m able to trust cause he used to do this work before he came here. He was very experienced and knew what to do.” Several participants stated they worked toward going back to school or seeking employment because their youth workers pushed them or believed in them more than they felt other adults in their lives had. One youth stated, “When you’re in a bad position or feeling down, you don’t have that motivation…they are always right there and it’s like you have this opportunity and we can do this.”

Building supportive relationships with program staff countered the most common barrier reported by youth to engaging in services, which was hesitation around trusting staff. One participant said, “I thought…I didn’t really vibe with professional people because I feel like they are not down to earth. I felt like I had to put on a mask with these people with all smiles…that’s why I wasn’t open to help at all from anyone.”

*Social Activities.* Staff at two funded agencies mentioned social activities as engagement strategies. Staff at one agency said the “selling point” for engaging youth in programming was “summer trips.” Participants were eager to be involved in the program so that they could build community with the other participants and participate in these trips and other social activities.

Participants also frequently mentioned social activities as key to program engagement. Youth from two agencies stated that field trips kept them interested in attending the program, and participants from another agency suggested increasing the number of social activities the program provides. When asked how to make the program better, one participant stated, “More playdates cause I don’t really know that many people around here…It would be nice to have more parent get-togethers cause sometimes when you’re a young parent you don’t go out as much.”

*Support Groups.* The opportunity to interact with other adolescent parents was an important aspect of support groups. When discussing a parenting group offered, one participant said,

The other thing about the groups, too, is that sometimes there is support within the groups, like she can teach me something that I didn’t know. It’s like we’re all the same age, we can relate to each other, we come from the same background, same city, same everything.

Some participants enjoyed the groups primarily for the social aspects of connecting with other adolescent parents. When asked about their favorite part of the program, one participant said, “Groups. Cause it was like fun…we used to come every week. We were having so much fun.”

Two participants stated not liking groups: one preferred one-on-one interaction with program staff and another preferred smaller groups, stating that the groups offered had too many people in them. Several other participants described not being open to groups when beginning the program because they did not feel comfortable, but that after some time they opened up and enjoyed the groups.

### Staffing Model

Another theme among both the staff and participants was staffing. Building time into staff schedules to coordinate case management with other program staff appeared to be critical to clearly communicating with participants. Four agencies used a centralized staffing model in which services were offered directly by the agency and participants were assigned a youth worker who acted as their point person. One agency used a network model in which multiple organizations provided direct services and participants typically did not have one point person. Program staff at two of the agencies with a centralized approach emphasized the importance of staff communication to coordinate care and suggested discussing every case at least once per month in a group setting. Program staff at the agency using the network model discussed staff communication as a significant challenge:

When you are trying to create a fluid system and everyone has their own system…[it] is a challenge to making sure that young people are being taken care of. Everyone is in their silo doing their own thing but you’re trying to cross refer.... Good vision, but I think there were too many cooks in the kitchen.

Youth participants in the network model also noted that communication and coordination of services were barriers: “Nobody communicated with me. Like…you got referred cause of this, this, and that. They didn’t tell me that…. Who referred me? So it was weird to meet all these people all at once and all saying that they were my [youth] worker.”

### Concrete Supports

All agencies reported that they offered incentives to increase program engagement. When probed, agencies defined incentives as supports such as child care, transportation, food, and clothing. No MPPTI agencies offered cash incentives for program participation. Youth reported that these supports allowed them to more easily access the program: “That was like the best thing when I first came here, when they said that they had the child care so it makes it easy.”

## Discussion

We identified three key strategies—social-emotional supports, staffing model, and concrete supports—perceived to be important in program engagement by MPPTI program staff and participants. Quantitative data on program retention coupled with qualitative data from staff and youth interviews suggest that in MPPTI, there may be an association between some of the strategies identified and levels of program retention. Much of the previous research on youth program engagement has not focused exclusively on adolescent parents and therefore may miss some of the unique needs of this population. Participants most frequently reported social-emotional supports as key to program engagement. To our knowledge, few studies examine social activities as engagement strategies for young parents. The link between providing social-emotional supports and in particular purely social activities such as field trips or play dates in relation to program retention and achieving program outcomes should continue to be explored.

Young parents in programs with more centralized staffing models described better communication and trusting relationships with program staff. This theme corresponds to previous research identifying quality staff-participant relationships as key to program engagement (Greene et al. 2013; Rhodes 2004). We hypothesize there may be an association between staffing model and the level of social-emotional support that participants experience via staff-participant relationships. Future studies could examine whether having a single point of agency contact per participant leads to more supportive staff relationships and greater levels of program engagement.

While the two agencies with the highest levels of program retention used a “centralized” model and offered all engagement strategies we identified, a third agency employing all engagement strategies identified and using a centralized staffing model did not have a similar level of program retention. Our analysis does not capture all of the program components or other factors related to program engagement. For example, social activities were identified as a program component by three agencies, but we did not examine the types of social activities offered by agency, the frequency with which activities were offered, or how many youth participated in each type of activity. Other program components such as the quality of programming related to employment or educational supports were not included in our study and may also play an important role in program engagement.

Spearman correlation results indicated significant associations between homelessness and not being in school/not being employed at program entry and program retention. The two agencies with the highest percentages of homeless participants at program entry also had the lowest levels of program retention. Our study did not explore the interaction between participant characteristics and engagement strategies in relation to program retention. Some engagement strategies may be more effective for certain populations. Further research could examine which engagement strategies are most effective, especially within the realm of social-emotional supports.

There are limitations to this study. Youth focus groups were conducted among a convenience sample of current program participants over the age of 18. Therefore, the views expressed may not represent all youth in programming and may differ from the views of participants under age 18 and participants who were no longer engaged in the program at the time of the focus groups. We examined only five sites which differed on participant characteristics, location, and other factors beyond the engagement strategies and staffing model we examined in this paper. We cannot rule out that differences in these factors may be predictors of program engagement because we did not conduct robust analyses on these factors. Finally, we did not measure the quality of the engagement strategies identified or of the staff relationships with participants.

This paper builds upon previous research identifying specific strategies for youth program engagement (Asheer et al. 2014; Greene et al. 2013; Rhodes 2004). In addition to offering concrete supports and emphasizing quality staff-participant relationships, programs seeking to engage adolescent parents should consider the social isolation that many young parents experience and build a variety of social-emotional supports into their programs. Strategies for staff retention and training to ensure continuous care and to maintain trusting relationships between program participants and staff may also be beneficial. To identify which engagement strategies and program models may be most effective for specific populations, future research could explore how participant characteristics may be related to program model and engagement. Relationships between program content, social-emotional supports, and staff relationships could be examined to develop more effective programming for adolescent parents and their children. Focusing this research on adolescent parents may help meet the unique needs of young families.

## Appendix

See Table

**Table 4:** Comparison of program retention by MPPTI site, 2014–2017

Site pair	Difference between means in months	95% confidence limits	Significant at 0.05 level
Chelsea–Lawrence	2.2	− 2.5, 6.8	
Chelsea–New Bedford	6.5	1.2, 11.8	*
Chelsea–Holyoke	7.1	2.1, 12.1	*
Chelsea–Springfield	9.6	4.9, 14.4	*
Holyoke–Chelsea	− 7.1	− 12.1, − 2.1	*
Holyoke–Lawrence	− 4.9	− 9.5, − 0.3	*
Holyoke–New Bedford	− 0.5	− 5.8, 4.7	
Holyoke–Springfield	2.6	− 2.2, 7.3	
Lawrence–Chelsea	− 2.2	− 6.8, 14.4	
Lawrence–New Bedford	4.4	− 0.5, 9.3	
Lawrence–Holyoke	4.9	0.3, 9.5	*
Lawrence–Springfield	7.5	3.1, 11.8	*
New Bedford–Chelsea	− 6.5	− 11.9, − 1.2	*
New Bedford–Lawrence	− 4.4	− 9.2, 0.5	
New Bedford–Holyoke	0.5	− 4.7, 5.8	
New Bedford–Springfield	3.1	− 2.0, 8.2	
Springfield–Chelsea	− 9.6	− 14.4, − 4.6	*
Springfield–Lawrence	− 7.5	− 11.8, − 3.1	*
Springfield–New Bedford	− 3.1	− 8.2, 2.0	
Springfield–Holyoke	− 2.6	− 2.6, − 7.3	

*Notes.* Pairwise comparisons made using Scheffe’s test. *MPPTI* Massachusetts Pregnant and Parenting Teen Initiative

4

## References

[CR1] Angley M, Divney A, Magriples U, Kershaw T (2015). Social support, family functioning and parenting competence in adolescent parents. Maternal and Child Health Journal.

[CR2] Asheer S, Berger A, Meckstroth A, Kisker E, Keating B (2014). Engaging pregnant and parenting teens: Early challenges and lessons learned from the evaluation of adolescent pregnancy prevention approaches. Journal of Adolescent Health.

[CR3] Assini-Meytin L, Greene KM (2015). Long-term consequences of adolescent parenthood among African-American urban youth: A propensity score matching approach. Journal of Adolescent Health.

[CR4] Crawford DM, Trotter EC, Sittner Hartshorn KJ, Whitbeck LB (2011). Pregnancy and mental health of young homeless women. American Journal of Orthopsychiatry.

[CR5] Ellis-Sloan K, Tamplin A (2019). Teenage mothers and social isolation: The role of friendship as protection against relational exclusion. Social Policy and Society.

[CR6] Elo S, Kyngas H (2008). The qualitative content analysis process. Journal of Advanced Nursing.

[CR7] Gneezy U, Meier S, Rey-Biel P (2011). When and why incentives (don’t) work to modify behavior. Journal of Economic Perspectives.

[CR8] Greene KM, Lee B, Constance N, Hynes K (2013). Examining youth and program predictors of engagement in out-of-school time programs. Journal of Youth and Adolescence.

[CR9] Harrison ME, Clarkin C, Rohde K, Worth K, Fleming N (2017). Treat me but don’t judge me: A qualitative examination of health care experiences of pregnant and parenting youth. Journal of Pediatric Adolescent Gynecology.

[CR10] Ickovics JR, Earnshaw V, Lewis JB, Kershaw TS, Magriples U, Stasko E, Tobin JN (2016). Cluster randomized controlled trial of group prenatal care: Perinatal outcomes among adolescents in New York City health centers. American Journal of Public Health.

[CR11] Jacobs F, Easterbrooks MA, Goldberg J, Mistry J, Bumgarner E, Raskin M, Fauth R (2016). Improving adolescent parenting: Results from a randomized controlled trial of a home visiting program for young families. American Journal of Public Health.

[CR12] Lanzi RB, Bert SC, Jacobs BK (2009). Depression among a sample of first time adolescent and adult mothers. Journal of Child and Adolescent Psychiatric Nursing.

[CR13] Lipman EL, Georgiades K, Boyle M (2011). Young adult outcomes of children born to teen mothers: Effects of being born during their teen or later years. Journal of the American Academy of Child and Adolescent Psychiatry.

[CR14] Logsdon MC, Gagne P, Hughes T, Patterson J, Rakestraw V (2005). Social support during adolescent pregnancy: Piecing together a quilt. Journal of Obstetric, Gynecologic, & Neonatal Nursing.

[CR15] Malabarey OT, Balayla J, Klam SL, Shrim A, Abenhaim HA (2012). Pregnancies in young adolescent mothers: A population-based study on 37 million births. Journal of Pediatric Adolescent Gynecology.

[CR16] Martin, J. A., Hamilton, B. E., Osterman, M. J. K., Driscoll, A. K., & Drake, P. (2018) Births: Final data for 2016*. National Vital Statistics Reports, 67*(1)*.* Retrieved from https://www.cdc.gov/nchs/data/nvsr/nvsr67/nvsr67_01.pdf 29775434

[CR17] Massachusetts Department of Public Health. (2018). Massachusetts births 2016*.* Retrieved from https://www.mass.gov/files/documents/2018/06/01/birth-report-2016.pdf

[CR18] Recto P, Champion JD (2018). “We don’t want to be judged”: Perceptions about professional help and attitudes towards help-seeking among pregnant and postpartum Mexican-American adolescents. Journal of Pediatric Nursing.

[CR19] Reid V, Meadows-Oliver M (2007). Postpartum depression in adolescent mothers: An integrative review of the literature. Journal of Pediatric Health Care.

[CR20] Rhodes JE (2004). The critical ingredient: Caring youth-staff relationships in after-school settings. New Directions for Youth Development.

[CR21] Tong A, Sainsbury P, Craig J (2007). Consolidated criteria for reporting qualitative research (COREQ): A 32-item checklist for interviews and focus groups. International Journal for Quality in Health Care.

